# Immune parameters associated with survival in metaplastic breast cancer

**DOI:** 10.1186/s13058-020-01330-6

**Published:** 2020-08-18

**Authors:** Xue Chao, Lili Liu, Peng Sun, Xia Yang, Mei Li, Rongzhen Luo, Yuhua Huang, Jiehua He, Jingping Yun

**Affiliations:** 1 Sun Yat-sen University Cancer Center, State Key Laboratory of Oncology in South China, Collaborative Innovation Center for Cancer Medicine, Guangzhou, People’s Republic of China; 2 Department of Pathology, Sun Yat-sen University Cancer Center, 651 Dongfeng East Road, Guangzhou, 510120 People’s Republic of China

**Keywords:** Metaplastic breast cancer, Immune characteristics, Prognosis

## Abstract

**Background:**

Metaplastic breast carcinoma (MBC) is a rare histological type of breast cancer, which commonly shows resistance to standard therapies and is associated with poor prognosis. The immune microenvironment in MBC and its significance has not been well established due to its low incurrence rate and complex components. We aimed to investigate the diversity of immune parameters including subsets of TILs and PDL1/PD1 expression in MBC, as well as its correlation with prognosis.

**Methods:**

A total of 60 patients diagnosed with MBC from January 2006 to December 2017 were included in our study. The percentage (%) and quantification (per mm^2^) of TILs and presence of tertiary lymphoid structures (TLS) were evaluated by hematoxylin and eosin staining (HE). The quantification of CD4+, CD8+ TILs (per mm^2^), and PD-1/PDL1 expression were evaluated through immunohistochemistry and analyzed in relation to clinicopathological characteristics. A ≥ 1% membranous or cytoplasmatic expression of PD1 and PDL1 was considered a positive expression.

**Results:**

We found squamous cell carcinoma MBC (33/60, 55%) exhibiting most TILs of all the MBC subtypes (*p* = 0.043). Thirty-three of 60 (50%) of the patients had coexisting invasive ductal carcinoma of no special type (IDC-NST), and the average percentage of TILs in MBC components was lower compared with NST components (*p* < 0.001). Thirty (50%) patients exhibited positive (≥ 1%) PDL1 expression in their tumor cells, while 36 (60%) had positive (≥ 1%) PDL1 expression in their TILs. Twenty-seven (45%) of all the patients had positive (≥ 1%) PD1 expression in their tumor cells and 33 (55%) had PD1-positive (≥ 1%) stromal TILs. More CD8+ TILs were associated with positive PDL1 expression of tumor cells as well as positive PD1 expression in stromal cells. Greater number of stromal TILS (> 300/mm^2^, 20%), CD4+ TILs (> 250/mm^2^), and CD8+ TILs (> 70/mm^2^) in MBC were found associated with longer disease-free survival. Positive expression of PDL1 in tumor cells (≥ 1%) and PD1 in stromal cells (≥ 1%) were also associated with longer survival.

**Conclusions:**

The immune characteristics differ in various subtypes as well as components of MBC. Immune parameters are key predictive factors of MBC and provide the clinical significance of applying immune checkpoint therapies in patients with MBC.

## Background

Metaplastic breast carcinoma (MBC) is a rare subtype of invasive breast carcinoma, which accounts for approximately 1% of all breast malignancies [1]. MBC displays various histological subtypes, exhibiting metaplastic change from neoplastic epithelium to squamous cells and/or mesenchymal elements. MBC usually lacks the expression of hormone receptor and HER2 and is considered as a subtype of triple-negative breast cancer (TNBC). Previous studies have shown that MBC is less sensitive to adjuvant therapy [2, 3] and has poorer prognosis compared with invasive ductal carcinoma in the same clinical stage [4, 5], including the TNBC [6].

Tumor-infiltrated lymphocytes (TILs) are mononuclear lymphocytes present in the tumoral tissue, reflect an immune response in the tumor microenvironment, and can be easily identified in formalin-fixed paraffin-embedded tissues. Increased TILs in tumors were found to be associated with better prognosis and an increase in systematic therapy sensitivity in TNBC [7–9]. The subsets of TILs were also shown to be a prognostic factor for TNBC [10, 11]. The dynamic expression of programmed cell death ligand 1 (PDL1) and PD1 on both the tumor and immune cells can either disrupt or sustain tumor growth, which is correlated with prognosis in breast cancer [12–14]. Furthermore, the TILs and PDL1/PD1 expression were both predictors of effectiveness of immune checkpoints therapy in breast cancer [15, 16] and TILs were shown to be correlating with PDL1/PD1 expression [17].

Data involving the immune microenvironment of MBC is limited and short of quantification. Given the heterogeneous components of MBC and the prognostic significance of TILs and its subsets as well as PDL1/PD1 expression in breast cancer, we investigated the expression of PD1/PDL1 and quantified TILs to determine their association with clinicopathological features and survival outcome in a cohort of MBC.

## Methods

### Patients and samples

This study was conducted using the data of patients diagnosed with MBC from January 2006 to December 2017, treated at the Sun Yat-sen University Cancer Center. All patients diagnosed with MBC were reviewed. Patients with recurrences at diagnosis, previous malignancies, and immune deficiencies were excluded. The clinical parameters investigated were age, pathological diagnosis, symptoms, present history, past history, image examination including ultrasound, and mammography results, operative records, and adjuvant therapy data were extracted from the original medical records. The follow-up information was gained from medical records and telephonic interviews. The primary endpoint of the study was disease-free survival. The protocol of this study was approved by the institutional Ethics Committee of Sun Yat-sen University Cancer Center, and consent for the use of data in research was obtained from each participant.

### Pathological assessment

The pathological categories of MBC were considered based on the WHO classification [18]. The subtypes included low-grade fibromatosis-like carcinoma, squamous cell carcinoma, spindle cell carcinoma, and carcinoma with mesenchymal differentiation (chondroid, osseous, and other mesenchymal differentiation). A mixed type MBC was considered when 2 or more subtypes of MBC were present on the histological slides. All the original tumor slides of each patient were reviewed by 2 pathologists. The hormone receptor and HER2 receptor status were extracted from the original pathological reports.

### Evaluation of TILs

TILs were evaluated on the hematoxylin and eosin (H&E) sections of the tumor following the guidelines of the international TILs working group [19]. The TILs were evaluated within the invasive border and a percentage, as well as a quantification of TILs in square millimeter, was given. An average percentage and quantification of TILs were documented for each case. For mixed type or MBC with invasive ductal carcinoma of no-special type (IDC-NST), the TILs percentage was evaluated in different components, while the TILs quantification was counted as an average number. The stromal TILs were analyzed for the epithelial tumor component. For cases with mesenchymal compartment, the TILs in the epithelium were called intra-epithelium TILs, while the TILs analyzed within the mesenchymal element were called mesenchymal TILs. Tertiary lymphoid structure (TLS), aggregates that recapitulate the components and architecture of a lymph node, was also evaluated as previously described [19, 20]. Dual staining of CD3/CD20 was also performed to validate the TLS number according to Buisseret’s study [21]. Furthermore, the quantification of TILs was performed manually through the digital scan using the Aperio imagescope (Leica Biosystems). Two pathologists evaluated all the data above separately and blind to the clinical outcomes. Consensus was reached between the two authors if there was a discrepancy among the collected data.

### Immunohistochemical evaluations

Formalin-fixed paraffin-embedded (FFPE) tissue sections were stained for PD-L1 (clone: antihuman PD-L1 rabbit monoclonal antibody E1L3N, Cell Signaling Technology), PD-1 (Clone UMAB199, ZSGB-Bio), CD4 (Clone EP204, ZSGB-Bio), CD8 (Clone SP16, ZSGB-Bio), and CD68 (Clone PG-M1, ZSGB-Bio). Dual CD3/CD20 immunohistochemical stain was performed as Buisseret et al. [21] (Supplemental Figure 1). A ≥ 1% membranous or cytoplasmatic expression of PD1 and PDL1 in tumor cells was considered positive expression. An immune cell was considered “PD-L1/PD-1 positive” if it featured any PD-L1 staining due to the small size of the lymphocytes. The percentage of PD-L1-positive tumor cells was proportionally evaluated in all tumor cells. PD-1 and PD-L1 immune cells were assessed relative to the whole tumor area, and as previously described [14, 22]. Quantification of CD4 and CD8 positive TILs were performed with digital imaging analysis (Halo imaging analysis software; Indica Labs, Corrales, NM) as well as manually. We manually annotated the system to indicate different components of MBC including epithelial area, mesenchymal area, and the stromal area. The software counted the number of positive immune cells in the tumor areas of the whole slides while the two pathologists counted the positive immune cells through the digital scan of the slides separately. Consensus was reached between the two authors if there was a discrepancy among the collected data.

### Statistics

Categorical variables were grouped based on the clinical findings, and decisions on the groups were made before modeling. The results were compared using the *χ*^2^ test or Fisher’s exact test. Continuous variables were compared using the *t* test. Comparison of TILs parameters between different groups used Wilcoxon test due to the limited sample size. Spearman’s rank correlation tests were used to assess the associations among infiltrations of CD4+, CD8+TILs, and PD-L1+ tumor/immune cells. The median of the CD4+, CD8+, and overall TILs counts is used as a cut-off value. Cox regression models were used to examine the prognostic effect of each variable. Kaplan-Meier curves were used to compare subgroups defined by biomarkers. A *p* value < 0.05 was considered statistically significant. All statistical analyses were carried out using the SPSS software, version 25.0 (IBM Corp, 1987, Chicago, USA), and GraphPad Prism 8 (GraphPad software, Inc.).

## Results

### Clinicopathological characteristics

A total of 60 surgically resected FFPE MBC samples were assessed in our study. The median age at diagnosis was 50 years (range, 25–81 years). The clinicopathological characteristics are listed in Table 1. Of all the 60 MBC patients, 33 were diagnosed as squamous cell carcinoma, 4 as spindle cell carcinoma, 8 as mesenchymal differentiation, 2 as fibromatosis-like, and 13 as mixed type.

**Table 1:** Clinicopathological characteristics

Patients characteristics	***n***	%
**Age (median/range)**	**50/25–81**	
**Histological subtype**		
Squamous cell carcinoma	**33**	55.0
Spindle cell carcinoma	**4**	6.56
Chondriod differentiation	**4**	6.56
Osseous differentiation	**4**	6.56
Fibromatosis-like	**2**	3.28
Mixed type	**13**	21.67
With invasive carcinoma (no special type)	**33**	55.0
**Tumor size(mm)**		
0–20	**10**	16.67
21–50	**33**	55
> 50	**17**	28.33
**Lymph node metastasis**		
Negative	**41**	68.33
1–3	**17**	28.33
4–10	**2**	3.33
> 10	**0**	–
**Lymphovascular invasion**		
Present	**16**	26.7
Absent	**41**	68.3
Unknown	**3**	5
**Surgery type**		
Breast-conserving surgery	**7**	11.67
Mastectomy	**53**	88.67
**Systematic therapy**		
Chemotherapy*	**56**	93.33
Radiotherapy	**52**	86.67
**Clinical outcome**		
Local recurrences	**5**	8.02
Distant metastasis	**7**	11.48
Total death	**6**	9.81
Cancer-specific death	**3**	4.92

*4/56 patients had neoadjuvant chemotherapy

A mixed MBC was defined as two or more metaplastic components. For the 13 mixed MBCs, 4/13 were mixed with squamous cell carcinoma and chondroid/chondroid matrix production; 6/13 were mixed with squamous cell carcinoma, spindle cell type, and invasive carcinoma with no special type; and 3/13 were mixed with squamous cell carcinoma and spindle cell type.

Thirty-three of 60 of the patients had coexisting IDC-NST. Twenty-eight of 33 of these tumors were triple-negative while 5/33 were hormone receptor-positive. Additional details are listed in Supplemental Table 1.

### Immune characteristics of different subtypes of MBC

**Fig. 1:**
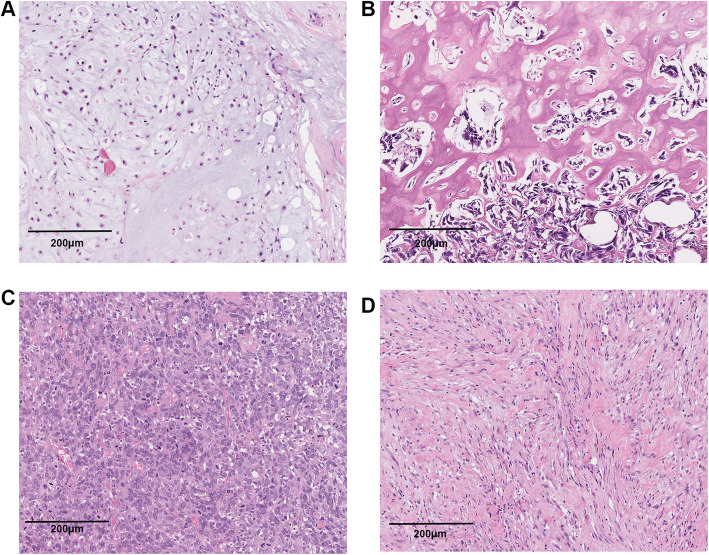
Hematoxylin and eosin-stained section of MBC with mesenchymal elements and the distribution patterns of TILs. **a** Chondroid differentiation. **b** Osseous differentiation. **c** Spindle cell. **d** Fibromotosis-like

For pure carcinoma, the TILs mainly located in stroma around the carcinoma nests. While for MBC with mesenchymal components, the TILs infiltrated diffuse around the mesenchymal cells. MBC with different mesenchymal elements were displayed in Fig. 1.

**Fig. 2:**
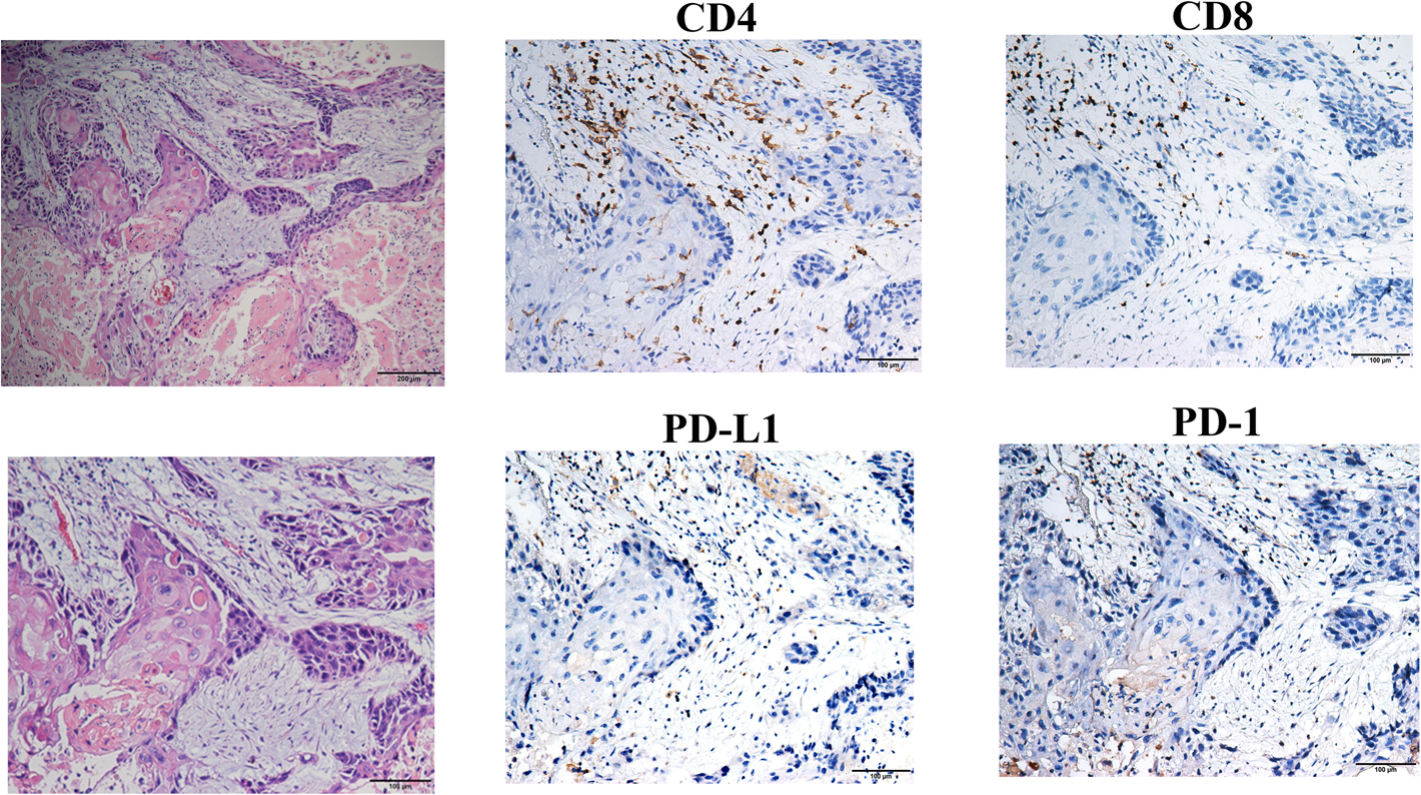
Representative hematoxylin and eosin-stained section of a mixed type MBC (squamous cell cancer component and chondroid-matrix component) with corresponding CD4, CD8, PDL1, and PD1 stains (original magnification × 200)

For each case, an average number of total TILs as well as CD4+ and CD8+ TILs, the percentage of TILs of each component, and the percentage of positive PD1 and PDL1 expression in both tumor cells and TIL was evaluated. Figure 2 showed a mixed type MBC composed of both squamous cell cancer and chondroid matrix and its expression of different immune parameters (CD4, CD8, PDL1, and PD1). The staining of CD68 in the same case was displayed in Supplemental Figure 2.

**Fig. 3:**
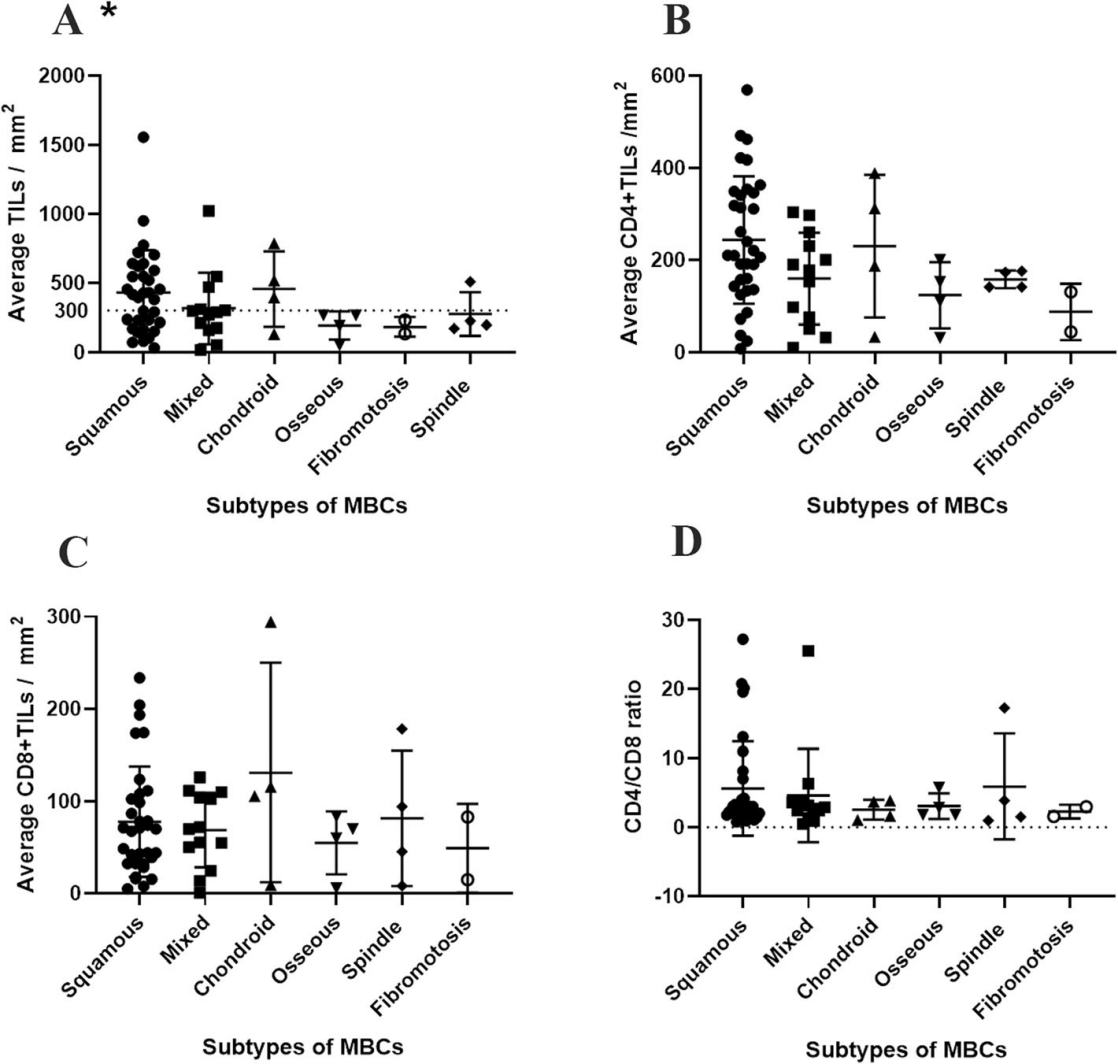
The stromal TILs counts in different MBC subtypes (**a**). The squamous cell carcinoma MBCs were more likely to have > 300/mm^2^ TILs (*p* = 0.043); CD4+ TILs counts in different MBC subtypes (**b**); CD8+ TILs counts in different MBC subtypes (**c**) and CD4/CD8 ratio in different MBC subtypes. The asterisk in the figure refers to *p* < 0.05

Of all subtypes of MBC, the squamous cell carcinoma MBC exhibited the greatest number of TILs. The squamous cell carcinoma MBCs were more likely to have > 300/mm^2^ (median) TILs compared to other subtypes (*p* = 0.043). None of this tendency was observed in CD4+ and CD8+ TILs. Neither does the CD4/CD8 ratio nor the CD68 positive cells displayed difference among MBC subtypes (Fig. 3 and Supplemental Figure 3).

**Fig. 4:**
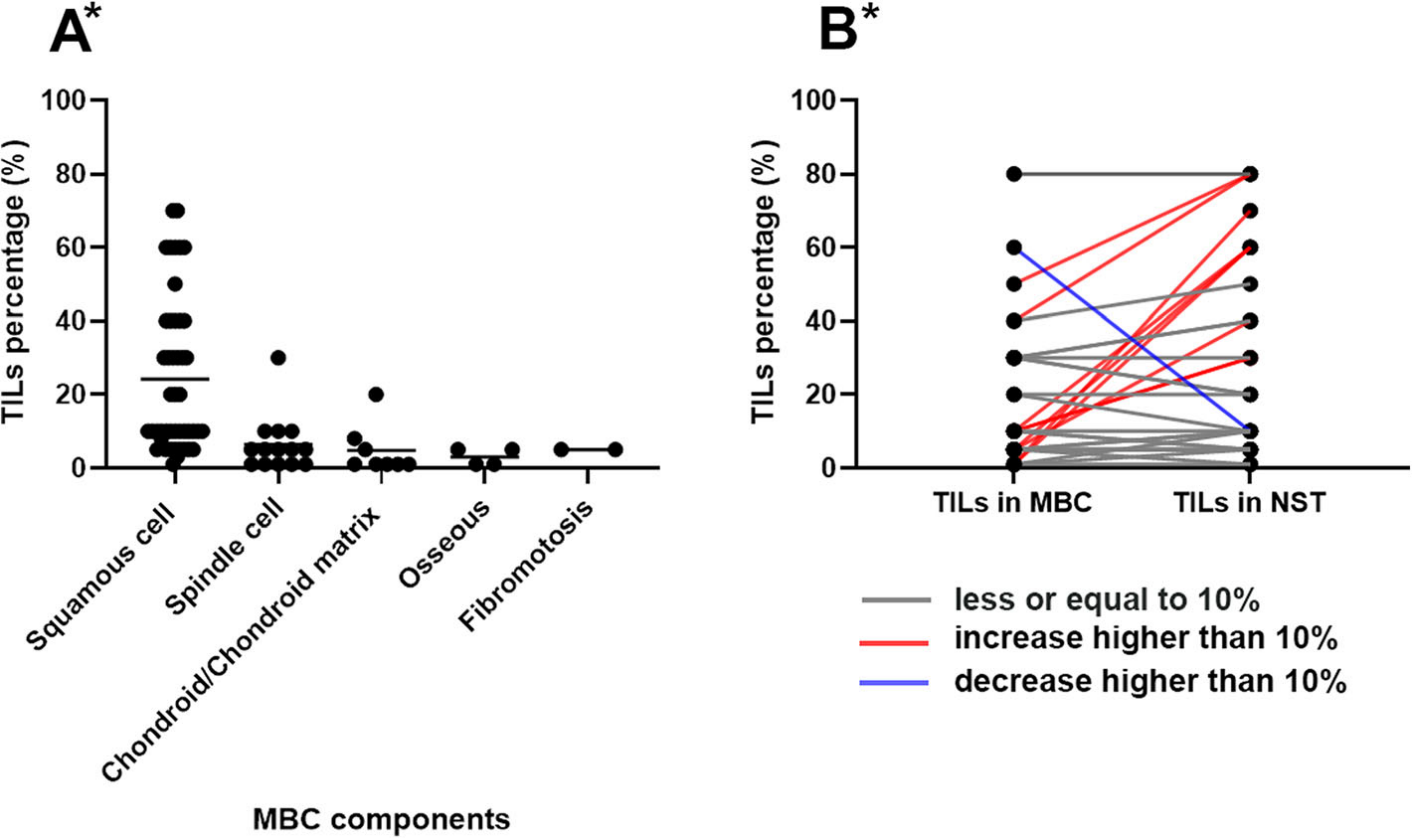
**a** The stromal TILs percentage in different MBC components. **b** Change of TILs between MBC components and accompanied invasive ductal carcinoma of no special type. Of all the 33 cases, increase in 9, no change in 23, and decrease in 1. TILs, tumor-infiltrating lymphocytes; MBC, metaplastic breast cancer; NST, no special type. The asterisk in the figure refers to *p* < 0.05

For different components of MBC, a comparison of TILs in percentage is shown in Fig. 4a. Of all the 5 components, squamous cells showed the most number of TILs (*p* < 0.001). For the 33 MBCs with IDC-NST, the average percentage of TILs in MBC components was lower in the IDC-NST components (Fig. 4b, *p* < 0.001). There is no difference in TILs percentage between simple MBC (27 cases) and MBC combined with IDC-NST (*p* = 0.25).

Thirty (50%) patients exhibited positive PDL1 expression in their tumor cells, while 36 (60%) had positive PDL1 expression in their TILs. Twenty-four (40%) patients had positive PDL1 expression in both tumor cells and TILs. Eighteen (30%) patients exhibited negative PDL1 in neither tumor cells nor TILs. Six (10%) patients had positive expression in tumor cells and negative expression in TILs. Twelve (20%) patients had positive expression in TILs and negative expression in tumor cells. Twenty-seven (45%) of all the patients had positive PD1 expression in their tumor cells and 24 (40%) had PD1 positive stromal TILs. The correlation of PDL1/PD1 expression in tumor cells and TILs were listed in Supplemental Table 2&3. There was no significant association of PDL1/PD1 expression between different MBC subtypes (Supplemental Table 4).

Among all the immune parameters, a higher level of CD8+ TILs was found to be correlated with positive PDL1 expression in both tumor cells and TILs (Table 2). Twenty-two patients had both greater number of CD8+ TILs (> 70/mm^2^) and positive PDL1 expression tumor cells.

**Table 2:** Relationship of PDL1 PD1 expression in tumors and stromal TILs with other immune parameters by (non-parametric) Spearman’s rank correlation

	PDL1 in tumor	PDL1 in stromal	PD1 in tumor	PD1 in stromal
TILs (> 300/mm^2^)	Rho 0.131	Rho 0.193	Rho 0.118	Rho 0.12
*p*	0.33	0.17	0.38	0.38
CD4 + TILs(> 250/mm^2^)	Rho 0.149	Rho 0.094	Rho 0.178	Rho 0.125
*p*	0.26	0.51	0.18	0.36
CD8+ TILs (> 70/mm^2^)	**Rho 0.492**	**Rho 0.367**	Rho −0.018	Rho 0.128
*p*	**< 0.001**	**0.006**	0.89	0.34
CD68+ TILs (> 300/mm^2^)	Rho 0.1	Rho − 0.053	Rho 0.018	Rho 0.015
*p*	0.45	0.71	0.89	0.91
TILs percentage (> 20%)	Rho − 0.034	Rho 0	Rho − 0.016	Rho − 0.165
*p*	0.80	1	0.42	0.22

### Univariate and multivariate analysis

We further evaluated the prognostic values of these immune parameters. There is a median follow-up of 48 months (range, 22–163 months).

**Fig. 5:**
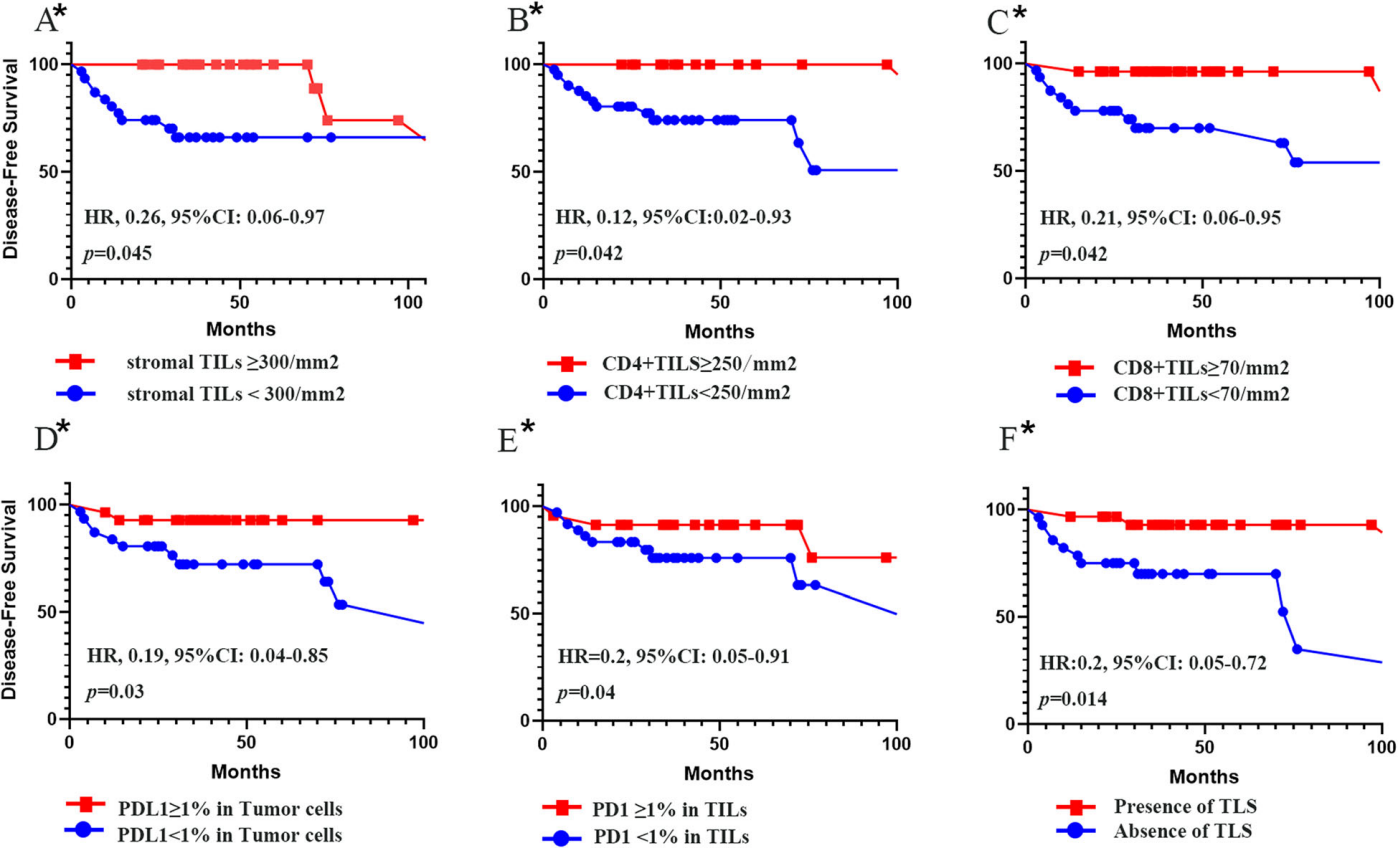
The prognostic values of immune parameters in MBC. **a** Stromal TILs. **b** CD4+ Stromal TILs. **c** CD8+ Stromal TILs. **d** PDL1 expression in tumor cells. **e** PD1 expression in stromal TILs. **f** Present of TLS in tumor. TILs, tumor-infiltrating lymphocytes; MBC, metaplastic breast cancer; TLS, tertiary lymphoid structure. The asterisk in the figure refers to *p* < 0.05

More TILs (cut-off ≥ 300/mm^2^) in patients with MBC showed a trend for better prognosis (HR, 0.26; 95%CI, 0.07–0.97; *p* = 0.045), and this trend become stronger in MBC with squamous cell cancer (HR, 0.07; 95%CI, 0.008–0.64; *p* = 0.018). Also, higher expression of CD4+ (cut-off ≥ 250/mm^2^) and CD8+ (cut-off ≥ 70/mm^2^) TILs keep this trend (CD4: HR, 0.12; 95%CI, 0.02–0.93; *p* = 0.042; CD8: HR, 0.21; 95%CI, 0.05–0.95; *p* = 0.042). Higher number of CD68-positive cells (> 300/m^2^) did not correlate with the patient’s outcome (HR 0.74, 95%CI 0.25–2.21). All the cut-off above was median of the data. The CD4/CD8 ratio had no correlation with DFS (HR, 0.95; 95%CI, 0.86–1.04; *p* = 27). Positive PDL1 expression in tumor cells (HR, 0.19; 95%CI, 0.04–0.85; *p* = 0.03) and PD1 expression in stromal TILs (HR, 0.20; 95%CI, 0.05–0.91; *p* = 0.04) also predicted a longer DFS. The presence of TLS in tumor predicted better prognoses as well (HR, 0.2; 95%CI, 0.05–0.75; *p* = 0.014) (Fig. 5).

In addition to the immune parameters described above, the lymphovascular invasion was also found to be significantly associated with shorter DFS (HR, 3.97; 95%CI, 1.19–13.23; *p* = 0.03). MBC histological subtypes, age, tumor size, lymph node status, and surgical type were not associated with DFS.

In multivariate analyses, only stromal TIL was identified as an independent indicator for DFS (HR, 0.17; 95%CI, 0.04–0.79; *p* = 0.02) (Table 3).

**Table 3:** Univariate and multivariate cox regression disease-free survival

Parameters	Univariate analysis		Multivariate analysis	
	HR (95%CI)	*p*	HR (95%CI)	*p*
**Age < 50 vs ≥ 50**	0.81 (0.27–2.41)	0.70	–	–
**Tumor size**				
0–20	1		–	–
21–50	1.03 (0.12–8.99)	0.98		
> 50	3.73 (0.74–32.06)	0.23		
**Lymph node status**				
Positive vs negative	0.92 (0.28–2.96)	0.88	–	–
**Lymphvascular invasion**				
Present vs absent	3.97 (1.19–13.23)	**0.03**	3.52 (0.89–13.89)	0.07
**With invasive ductal carcinoma (NST)**	0.98 (0.33–2.98)	0.97	–	–
**Surgery type**				
BCS vs mastecomy	1.07 (0.13–8.71)	0.95	–	–
**Histological subtype**				
Squamous cell	1	0.58		
Spindle cell	2.20 (0.25–19.50)			
Chondriod	1.36 (0.16–11.68)			
Osseous	4.44 (0.84–23.49)			
Fibromatosis-like	5.34 (0.61–47.10)			
Mixed type	1.31 (0.31–5.49)			
**Without/with NST**	1.05 (0.35–3.14)	0.93		
**Stromal TILs(/mm**^**2**^**)**				
**≥** 300 vs < 300	0.26 (0.07–0.97)	**0.045**	**0.17 (0.04–0.79)**	**0.024**
**CD4+ TILs(/mm**^**2**^**)**				
**≥** 250 vs < 250	0.12 (0.02–0.93)	**0.042**	0.99 (0.04–22.43)	0.99
**CD8+ TILs(/mm**^**2**^**)**				
**≥** 70 vs < 70	0.21 (0.05–0.95)	**0.042**	0.56 (0.11–2.94)	0.49
**Tumoral PDL1**				
Positive vs negative	0.19 (0.04–0.85)	**0.03**	0.39 (0.08–2.03)	0.26
**Stromal PD1**				
Positive vs negative	0.20 (0.05–0.91)	**0.04**	1.32 (0.26–6.87)	0.74
**TLS**				
Present vs absent	0.2(0.05–0.75)	**0.014**	0.61 (0.15–2.59)	0.51
TMIT* vs others	0.02 (0.01–2.53)	0.116		

NST, with invasive carcinoma (no special type)

*TMIT tumor microenviroment type refers to the higher CD8+ TILs and positive PDL1 expression of tumor cells

## Discussion

MBC is a rare subtype of TNBC with poor prognosis and usually not sensitive to conventional adjuvant therapy. Thus immune therapy may be a promising way to improve the outcome of MBC. In this study, we investigated 60 MBC samples for immune parameters associated with survival, searching for possible prognostic factors and treatment strategies.

MBC exhibited various histological features from epithelia to mesenchyme. Previous studies using whole-exome sequencing found that different components in MBC share identical somatic alterations [23]. Though different components of MBC shares the same origin, the immune characteristics vary a lot. To our knowledge, this is the first large study focusing on the different components of MBC to assess their immune microenvironment. We have observed that squamous cell MBCs were more likely to have > 300/mm^2^ TILs compared to other subtypes (*p* = 0.043). Also, squamous cell component displays the most TILs compared with other components (*p* < 0.001). For the 33 MBCs with IDC-NST, the average percentage of TILs in MBC components was lower in the MBC components compared with NST components. These results indicate that more epithelial differentiation, more TILs.

TILs are basic parameters reflecting the immune status of the tumor. The higher the expression of TILs has been associated with better survival in TNBC and HER-2 positive breast cancer, in both research and clinical practice. In MBC, more TILs were also proved to be correlated with longer DFS (HR, 0.26; 95%CI, 0.07–0.97; *p* = 0.045). The most of TILs are cytotoxic CD8+ T cells and CD4+ helper T cells. Both of these two groups play major parts in antitumor immunity [24]. CD8+ T cells can directly kill tumor cells. We found that more CD8+ TILs in MBC predicts better prognosis in MBC (HR, 0.21; 95%CI, 0.05–0.95; *p* = 0.042), which resonates with the findings of a previous study involving 1334 patients of all subtypes of breast cancer [25]. While another study found that the CD4+ TILs were independent prognostic factors in hormone negative breast cancer, no correlation was found between CD8+ TILs and clinical outcome [26]. Findings from the present study showed that CD4+ TILs correlated with better survival (HR, 0.12; 95%CI, 0.02–0.93; *p* = 0.042) as well as CD8+ TILs in MBC. TLSs are ectopic lymph node-like structures characterized by lymphoid aggregation with high endothelial venules. TLSs are induced in a chronic inflammatory environment and their presence is associated with the exacerbation of local immune responses [27]. Here, we found that the presence of TLS was associated with longer survival in MBC (HR, 0.2; 95%CI, 0.05–0.75; *p* = 0.014), as well as which were found in TNBC [28].

The expression of PDL1 in breast cancer has a controversial role in predicting prognosis of breast cancer. In the present study, the PDL1 expression in the tumor of MBC appeared in 50% (36/60) of all the patients, which is similar to the previously reported rate of 20%–58.5% in TNBC [14, 29, 30]. Our data suggested that PDL1 expression correlated with better survival (HR, 0.19; 95%CI, 0.04–0.85; *p* = 0.03) in MBC, which was consistent with some previous studies [31, 32] but contradicted with that of a meta-analysis involving 9 studies [33]. Different PDL1 immunohistochemistry assays, various scoring systems, and evaluation of different tumor compartments may be the reasons for this observed diversity [34]. Thus, a prospective study correlated with the treatment effect of PDL1/PD1 blockade is awaited to define the proper PDL1 expression assay.

The performance of different clones in PDL1 detection differs in breast cancer. PDL1 (E1L3N) identified more PD-L1-positive cases (14.7%, cut-off 1%) compared with SP142 (11.5%) and 28–8 (13.3%) in triple-negative breast cancer [35]. Adams et.al used PDL1 (E1L3N) detecting 62(48.75%) positive expression on tumor cells (cut-off 10%) in triple-negative breast cancer [36]. A meta-analysis of 38 studies revealed clone 28-8 yielding the highest positive rate (39%) on tumor cells in all breast cancer with different thresholds in all studies [37]. Data from IMpassion130 study using SP142 identified 369 (40.9%) PD-L1-positive expression on immune cells [38]. Our study used PDL1(E1L3N) identified 30 (50%) positive expression on tumor cells and 36 (60%) positive expression on immune cells, which may identify more PD-L1-positive tumors.

TILs infiltration pattern in MBC with mesenchymal elements differs from carcinoma due to the histological characteristics. The TILs infiltrated diffusely in the mesenchymal elements. The distribution of CD4+ and CD8+ TILs also follows this trend. Previous studies involved TILs and its subtypes also correlated with survival in soft tissue sarcoma [39–41]. A study involved 47 leiomyosarcomas revealed an average number of 10.5 CD4+ TILs and 16.1 CD8+ TILs per high power filed (CD4 33.87/mm^2^, CD8 51.93/mm^2^) [42], which is lower than the average number in MBC with mesenchymal elements (CD4 209.99 ± 140.10/mm^2^, CD8 83.34 ± 78.09/mm^2^). Further, no significant difference in TILs was found among different mesenchymal subtypes (*p* = 0.30). Another study revealed different PDL1 expression in various sarcoma [43]. Overall, 6/14 MBC with mesenchymal elements had positive PDL1 expression, no significant difference was observed among mesenchymal subtypes (*p* = 0.83).

MBC displays aggressive biological behavior, which is not sensitive to adjuvant therapy. Thus, immune checkpoints therapy may be a promising treatment for MBC. Previous clinical trials demonstrated that patients with PDL1-positive cells and increased TILs have better response to immune therapy [15, 44]. The selection of proper patients for immune therapy is largely based on the expression of PDL1/PD1 and density of TILs. We also found that both of the PDL1 expression in tumor cells (rho 0.492, *p* < 0.001) and stromal TILs (rho 0.367, *p* = 0.006) had a strong correlation with CD8+ TILs number. And these results were as expected that CD8+ T cells may be more sensitive to modulation of PDL1/PD1 pathway [45]. Tumors with a greater number of CD8+ TILs and positive PDL1 expression tumor cells were identified as tumor microenvironment type (TMIT) 1 according to the previous study [46]. Twenty-two patients were identified as TMIT 1 in our study, who can benefit from anti-PDL1-PD1 therapy. Our results suggest that the combination of these immune parameters may help to improve MBC patient selections for immune therapy. Further, a study indicated the relationship between PDL1 expression and macrophages (CD68 positive) [47], while we did not observe the tendency in MBC.

In this study, we have evaluated the TILs and immune checkpoints parameters expression in different components of MBC. The percentage of TILs as well as counts of TILs was both studied. These immune parameters have also been proved to be correlated with survival in MBC. However, we still have some limitations in this study. The sample size was limited due to the low incidence of MBC. Although the immune characteristics of MBC are similar with those observed in triple-negative breast cancer, it needs to be further validated in other data sets. Also, the detection of PDL1 expression was not correlated with treatment effect. The antibody (E1L3N) we used for PDL1 detection has been used and proved to have the highest positive rate in triple-negative breast cancer [35, 48]; it was not correlated with any PDL1/PD1 blockade drugs now and maybe overestimating PD-L1-positive tumors compared with FDA approved PDL1(SP142).

## Conclusions

We have demonstrated the immune characteristics of different subtypes in MBC. TILs, CD4+ TILs, CD8+ TILs, and the presence of TLS were found to be correlated with better prognosis in MBC. The expression of PDL1 in tumor cells was also found to be correlated with CD8+ TILs and associated with longer survival. These data suggested that immune checkpoint therapy may be a promising treatment in a certain type of MBC.

## Supplementary information

**Additional file 1: Supplemental Figure 1.** Representative images of the tertiary lymphoid structures stained with CD3/CD20 (CD3 = T cells, brown; CD20 = B cells, red).

**Additional file 2: Supplemental Figure 2.** Representative tissue section of a mixed type MBC (squamous cell cancer component and chondroid-matrix component, the same case in Fig. 2) with CD68 stains (original magnification × 200).

**Additional file 3: Supplemental Figure 3.** CD68+ TILs counts in different subtypes of MBC.

**Additional file 4: Supplemental Table 1**. Pathological characteristics of 33 cases with invasive carcinoma of no special type. **Supplemental Table 2**. The correlation of PDL1 expression in both tumor and stromal cells. **Supplemental Table 3.** The correlation of PD1 expression in both tumor and stromal cells. **Supplemental Table 4.** The PDL1 and PD1 expression in both tumor and stromal cells shares no difference in all subtypes of the MBCs.

## Data Availability

The authenticity of this article has been validated by uploading the key raw data onto the Research Data Deposit public platform (www.researchdata.org.cn), with the approval RDD number as RDDA2020001424.
