# Supplementary figures and images for: Immune parameters associated with survival in metaplastic breast cancer
Source: Breast Cancer Res. 2020 Aug 18;22:92. doi: 10.1186/s13058-020-01330-6 (PMC7437173; doi:10.1186/s13058-020-01330-6)

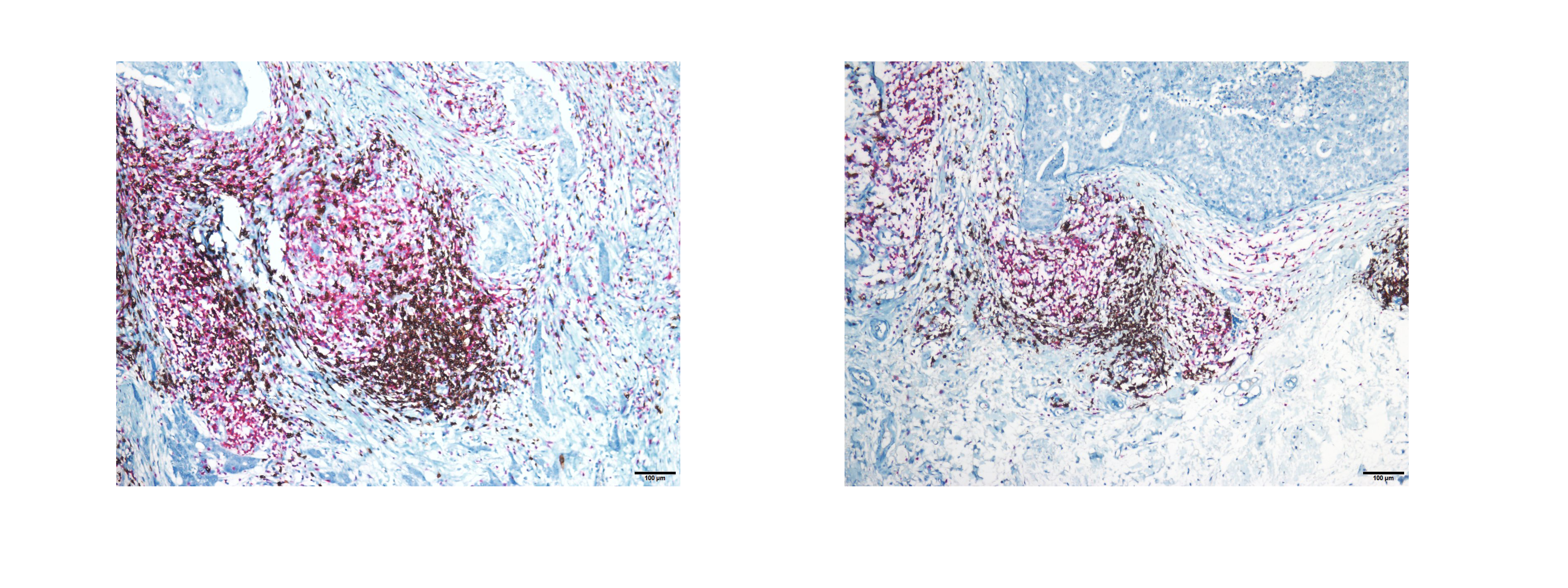

Supplement: Supplementary file 1 — Additional file 1: Supplemental Figure 1. Representative images of the tertiary lymphoid structures stained with CD3/CD20 (CD3 = T cells, brown; CD20 = B cells, red). [file 13058_2020_1330_MOESM1_ESM.tif]

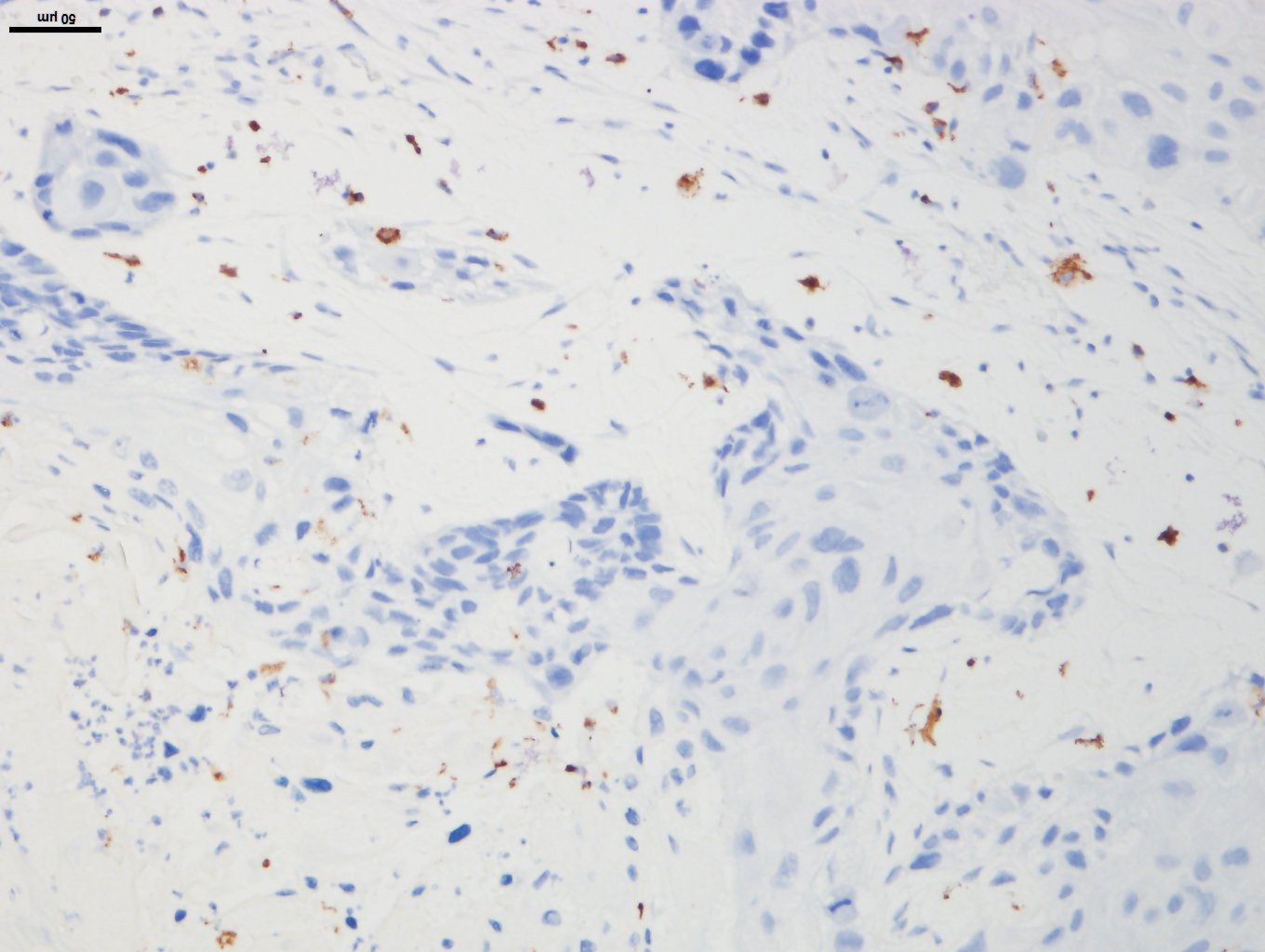

Supplement: Supplementary file 2 — Additional file 2: Supplemental Figure 2. Representative tissue section of a mixed type MBC (squamous cell cancer component and chondroid-matrix component, the same case in Fig. 2) with CD68 stains (original magnification × 200). [file 13058_2020_1330_MOESM2_ESM.tif]

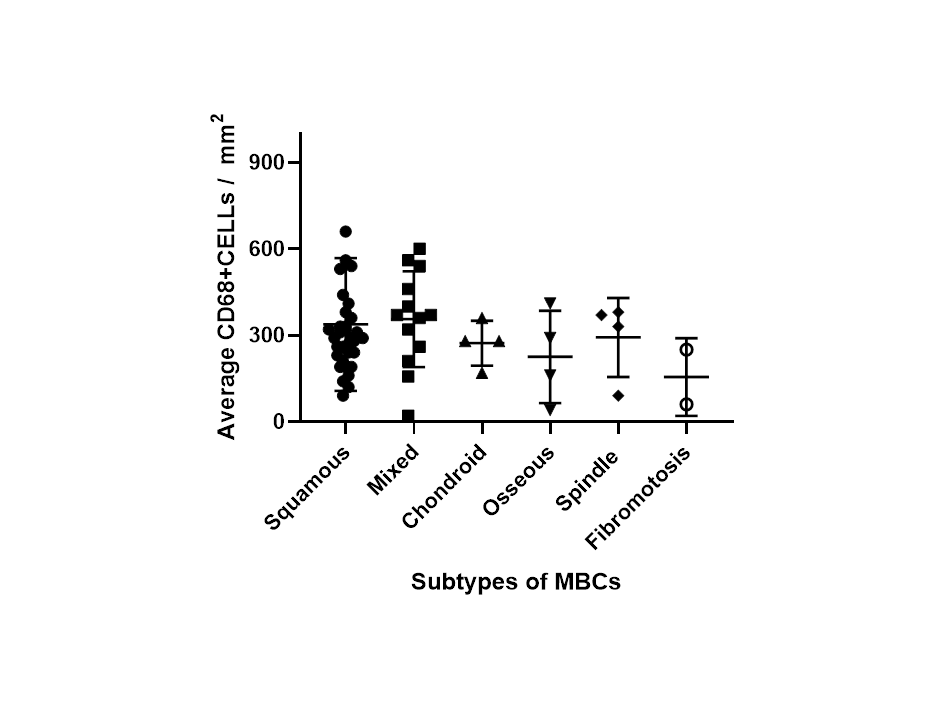

Supplement: Supplementary file 3 — Additional file 3: Supplemental Figure 3. CD68+ TILs counts in different subtypes of MBC. [file 13058_2020_1330_MOESM3_ESM.tif]
