# Supplementary material for: Immune parameters associated with survival in metaplastic breast cancer
Source: Breast Cancer Res. 2020 Aug 18;22:92. doi: 10.1186/s13058-020-01330-6 (PMC7437173; doi:10.1186/s13058-020-01330-6)
Supplement: Supplementary file 4 — Additional file 4: Supplemental Table 1. Pathological characteristics of 33 cases with invasive carcinoma of no special type. Supplemental Table 2. The correlation of PDL1 expression in both tumor and stromal cells. Supplemental Table 3. The correlation of PD1 expression in both tumor and stromal cells. Supplemental Table 4. The PDL1 and PD1 expression in both tumor and stromal cells shares no difference in all subtypes of the MBCs. [file 13058_2020_1330_MOESM4_ESM.docx]

**Supplemental Table 1 Pathological characteristics of 33 cases with invasive carcinoma of no special type**

| Pathological characteristics | N | % |
| --- | --- | --- |
| Histological grade |  |  |
| 2 | 1 | 3.03 |
| 3 | 32 | 96.97 |
| Hormone status |  |  |
| Positive | 5 | 15.15 |
| Negative | 28 | 84.84 |
| HER-2 status |  |  |
| Positive | 0 | 0 |
| Negative | 33 | 100 |
| Subtype |  |  |
| HR+/HER2- | 5 | 15.15 |
| TN | 28 | 84.84 |

**Supplemental Table 2 The correlation of PDL1 expression in both tumor and stromal cells.**

|  | PDL1 positive  in tumor cells | PDL1 negative  in tumor cells | Overall |
| --- | --- | --- | --- |
| PDL1 positive  in stromal cells | 24 | 12 | 36 |
| PDL1 negative  in stromal cells | 6 | 18 | 24 |
| Overall | 30 | 30 | 60(p=0.002) |

**Supplemental Table 3 The correlation of PD1 expression in both tumor and stromal cells.**

|  | PD1 positive  in tumor cells | PD1 negative  in tumor cells | Overall |
| --- | --- | --- | --- |
| PD1 positive  in stromal cells | 16 | 8 | 24 |
| PD1 negative  in stromal cells | 11 | 25 | 36 |
| Overall | 27 | 23 | 60(p=0.006) |

**Supplemental Table 4 The PDL1 and PD1 expression in both tumor and stromal cells shares no difference in all subtypes of the MBCs.**

| Subtypes | PDL1 in tumor(%)  (%) | PDL1in stroma  (%) | PD1 in tumor  (%) | PD1 in stroma (%) |
| --- | --- | --- | --- | --- |
| Squamous |  |  |  |  |
| <1% | 16(48.5) | 11(34.4) | 18(54.5) | 18(54.5) |
| ≥1% | 17(51.5) | 21(65.6) | 13(45.5) | 13(45.5) |
| Spindle |  |  |  |  |
| <1% | 1(25) | 2(50) | 2(50) | 2(50) |
| ≥1% | 3(75) | 2(50) | 2(50) | 2(50) |
| Chondroid |  |  |  |  |
| <1% | 1(25) | 1(25) | 3(75) | 3(75) |
| ≥1% | 3(75) | 3(75) | 1(25) | 1(25) |
| Osseous |  |  |  |  |
| <1% | 3(75) | 2(50) | 3(75) | 3(75) |
| ≥1% | 1(25) | 2(50) | 1(25) | 1(25) |
| Fibromatosis-like |  |  |  |  |
| <1% | 1(50) | 2(100) | 1(50) | 2(100) |
| ≥1% | 1(50) | - | 1(50) | - |
| Mixed |  |  |  |  |
| <1% | 7(53.8) | 4(30.7) | 7(53.8) | 7(53.8) |
| ≥1% | 6(46.2) | 9(69.3) | 6(46.2) | 6(46.2) |
| Total |  |  |  |  |
| <1% | 30(50) | 36(60) | 27(45) | 33(55) |
| ≥1% | 30(50) | 24(40) | 33(55) | 27(45) |
| ***p*** | 0.788 | 0.293 | 0.94 | 0.85 |
